# Eye–hand strategies in copying complex lines

**DOI:** 10.1016/j.cortex.2007.12.012

**Published:** 2009-03

**Authors:** John Tchalenko, R. Chris Miall

**Affiliations:** a Camberwell College of Arts, University of the Arts London, UK; b Behavioural Brain Sciences, School of Psychology, University of Birmingham, UK

**Keywords:** Drawing, Copying, Eye movements, Eye–hand interaction, Visuomotor

## Abstract

Eye movements and eye–hand interactions have been recorded for 10 beginner art students copying complex lines representing outlines of caricature heads seen in profile. Four copying conditions mimicking real-world drawing situations were tested: Direct copying where the original and copy were placed side by side, Direct Blind copying where the subject could not see the drawing hand and copy, Memory copying where the original was first memorized for drawing and subsequently hidden before drawing commenced, and Non-specific Memory copying where the original was encoded for facial recognition before being hidden and drawn from memory. We observed four very different eye–hand interaction strategies which provide evidence for the eye's dual role in the copying process: acquiring visual information in order to activate the visuomotor transformation and guiding the hand on the paper. The Direct copying strategies were best understood in terms of a Drawing Hypothesis stating that shape is the result of visuomotor mapping alone and, consequently, can be accurately drawn without vision of the drawing hand or paper. A double just-in-time mechanism is proposed whereby the eye refers alternatively to the original for shape and to the copy for spatial position just in time for the drawing action to proceed continuously.

## 1 Introduction

Artists drawing from life with their subject matter in front of them generally proceed detail by detail in a succession of short drawing episodes. Each episode is made of a gaze directed at the subject matter followed by a gaze directed at the paper, and in this way the eye is continually alternating between the two. During this time the hand transforms the three-dimensional scene of the external world into a two-dimensional picture on the paper. Copying is a special case of drawing from life where the scene itself is two-dimensional – a photograph, a painting or, as in the present study, a line drawn on paper. This line will be referred to as the “original”, and the drawing made thereof as the “copy”. A complex line is defined as one made up of a succession of simple lines, each being straight or of uniform curvature. We investigate here eye–hand strategies adopted in the copying of complex lines under different experimental conditions selected to mimic situations commonly found in real-world drawing situations.

The original lines used in this study represented the outlines of heads with caricatured features seen in profile. This shape was chosen to simplify the task for the subject by presenting a familiar succession of components (nose, mouth, chin, etc.) which, nevertheless, had to be carefully observed in order to record correctly their caricature aspects (exact shape of a pointed nose, protruding shape of the chin, etc.). Two types of test situations were examined: Direct copying where the original is online, i.e., constantly available to the subject's vision, and Memory copying where the original is withdrawn from view and a short time lapse introduced before drawing starts.

In drawing from life, each episode starts with the acquisition of visual information from the original. This information is transformed by the brain into a motor program, a process known as visuomotor mapping. The episode ends with the execution of the motor program in the form of a line drawn on the paper. To draw this line the hand holding the pencil must move along a path shaped exactly like the original line, with all points of the path relatively positioned to each other as they are on the original. The position qualifier is necessary to ensure one-to-one scaling. In simpler terms, copying is reproducing exactly shape and spatial position. In this task, the eye has the dual role of gathering visual information from the original and assisting the hand on the paper.

To our knowledge, eye–hand interactions during copying have not been previously documented, although a limited amount of data is available on the more general case of drawing from life. In particular, the frequency of gaze alternations between original and paper or canvas has been reported by several authors. Using the term cycle to define the time lapse between two references to the original, Miall and Tchalenko (2001) measured with a professional portrait artist Humphrey Ocean cycle rates between 12/min for a 5-h pencil portrait and 22/min for a 2-min pen sketch. Higher rates were measured by other authors: 25/min (Konecni, 1991), 28/min (Tchalenko et al., 2003), 35/min (Land, 2006) and 36/min (Cohen, 2005). However, the relationship between amount of drawing experience and cycle rate has never been specifically investigated, and in the present author's experience, there is at least as much variability between individuals of similar skills as between professionals and amateurs.

The location of fixations have also been studied in Humphrey Ocean's case: on the live model they were situated on the detail being captured, and on the drawing they were either located in the near vicinity of the pencil for short lines of 10–20 mm, or behind the pencil and following it with short saccades for longer lines (Miall and Tchalenko, 2001). Towards the end of a 5-h portrait, some long lines, drawn with great accuracy, were produced while the eyes were foveating entirely elsewhere on the paper or model, a behaviour which we will return to further on. This particular painter attached paramount importance to the precise shape and location of every line drawn. With other painters who draw more rapidly, or who use many short pencil markings from which the line emerges, single fixations can move around and along the line as it is being drawn (Tchalenko et al., 2003). Recently, Land (2006) documented another type of eye–hand coupling where the artist, drawing very quick 40-sec portraits, produced saccades from model to paper which brought fixations to what would become the end point of the line which was about to be drawn. Gowen and Miall (2006) observed a similar behaviour with subjects drawing squares. They found that fixations were often made at the corners of the square where the eye would remain until the pen tip moved to within approximately 1° of the eye position. Saccades were then made to a new location along the next side or to the next corner before the hand had reached the previous corner, a procedure cited as evidence for predictive hand control.

Of immediate relevance to the present study is the data on eye–hand interactions observed when drawing simple lines of straight or uniform curvature, i.e., where the shape is so simple that instructions to draw can be given verbally rather than by showing an example to copy (Tchalenko, 2007). Two dominant modes of eye–hand interaction were observed for straight and curved horizontal and vertical lines, lines between two predetermined points and lines defining a square. In the first, *close pursuit*, fixations kept up with the drawing hand, generally following but occasionally preceding the pencil tip with short saccades, only rarely locking onto the pencil tip in smooth pursuit. In the second, *target locking*, a stable fixation was held on the line's future end point throughout the drawing action. Close pursuit was found in situations were the action was led by the hand, and target locking, where the action was led by the eye. Depending on the type of line, subjects used one of these modes, or a specific combination of both modes, regardless of previous drawing experience. Both these fundamental modes will be encountered in the copying tests of the present study.

The basic assumption implicit in the studies of drawing from life mentioned above is that some form of working memory is involved in the drawing process. This stems from observations that when drawing while looking at the paper, the subject is not looking at the original and hence is presumably working from a visual memory representation (see for e.g., Phillips et al., 1978). This *conventional interpretation* posits the following sequence: the original, or part thereof, is first encoded to visual memory during fixation on the original, after which the subject turns to the paper and drawing proceeds from the stored mental image. As the image fades there comes a point where the subject needs to return to the original. Much of the eye tracker data obtained with Humphrey Ocean supported such an interpretation, but instances when this behaviour did not hold were also noted. In particular, Miall and Tchalenko (2001) and Tchalenko et al. (2003) described long complex lines drawn while the eye was foveating elsewhere on the picture as well as on the model itself. These lines were then reinforced very accurately, again without central vision. The only times the eye and hand coincided were at the starting point of the line when first drawn, and at the starting and ending point when the line was reinforced. The action was not one of tracing which is generally associated with eye movements of the smooth pursuit type (Gowen and Miall, 2006). Nor was it likely to be using parafoveal vision as the eyes were foveating precise details elsewhere, including on the model itself which was out of parafoveal range. Because in these cases the hand's movements did not seem controlled by the eye, the question arose whether drawing of the line was making use of some form of motor memory.

Other eye–hand interaction results which could not be adequately explained by the conventional model came from systematic eye movement tests while drawing simple lines (Tchalenko, 2007). For example, when the task was changed from straight to curved lines when drawing from a given point A to a given point B, the characteristic eye–hand interaction strategy adopted by most subjects did not alter. It seemed as if the shape of the line itself was “known” to the hand, and that the eye's role was restricted to ensuring that the line started at A and ended at B, i.e., a role of spatial positioning. Intuitively we know that a simple shape, such as a big S, can be successfully drawn blind without the eye seeing either the paper or the hand. However, if the drawing is to be of a given size, proportion or inclination, or if it is to start or end at predetermined points, then the paper must be seen as the line is being drawn.

These and other investigations in preparation on well-known artists drawing portraits suggest an additional way of drawing from life governed by a different eye–hand interaction principle, namely that the shape of the line to be drawn is acquired by the hand during the time that the subject is still looking at the original. In other words the visual information captured from the original is transformed into a motor programme that can be executed instantly, online, rather than retained as a mental image to be executed later after the subject has turned to the paper. Consequently, the role of the eye when the subject does turn to the paper is essentially one of spatial positioning. A *Drawing Hypothesis* may therefore be stated as follows: the drawing of shape is the result of visuomotor mapping that can be executed directly while perceiving the original and without vision of the drawing surface. The corollary to the hypothesis is that correct spatial positioning on the paper requires vision of the drawing surface. The difference between the two ways of drawing lies in the timing of the visuomotor mapping stage as schematized in Table 1.

These two ways of drawing are not mutually exclusive. Depending on the artist, on the type of drawing and, as described in the Ocean example, on the stage of the drawing, one may prevail over the other. The eye–hand interaction strategies investigated in the present study provided the opportunity for evaluating further the argument for the Drawing Hypothesis in the case of copying complex lines.

## 2 Experimental methodology and procedure

Subjects were seated 50 cm away from a vertical easel on which was mounted an A2 sheet of paper. At this distance 1° visual angle covers just under 10 mm on the paper. Drawing was with a soft lead pencil and head movements were unrestricted. The copy paper was placed next to the original on the vertical easel in front of the seated subject. The subject's eye and hand movements were recorded in the following situations.

### 2.1 Experiment 1: Direct copying

The original was placed just left-of-centre on the easel. The subject was instructed to draw an accurate copy immediately to the right of the original.

### 2.2 Experiment 2: Direct Blind copying

The original was placed centrally on the easel and the subject was given a sketch pad to hold on his/her lap. The subject was instructed to copy the original without looking at the sketch pad.

### 2.3 Experiment 3: Memory copying

The subject was instructed to memorize the original in order to subsequently draw it from memory. The original was displayed on the easel just left-of-centre for 8 sec. It was then covered up and after 10 sec the subject started drawing immediately to the right of the hidden original.

### 2.4 Experiment 4: Non-specific Memory copying

This was performed before all the other tests and before subjects were told that they would be asked to draw. A flipchart system displayed original heads, one by one, on the vertical easel. Each head was shown for 8 sec. The only instruction given to the subject was to signal verbally as soon as a repeat was spotted. Head No. 2 was repeated as No. 8. All subjects identified the repeat almost instantly in less than 1 sec. Head No. 8 was then replaced by a blank paper, an operation performed in about 3 sec, and only then was the subject given a pencil and asked to draw this head. The term “non-specific” is used to indicate that memorizing had not taken place with the specific intention of drawing.

In the real world, drawing strategies depend as much on the artist's preferences as on the surrounding material situation. Direct copying and its blind variant are common when the subject matter is very near the paper or canvas. Memory copying is common when using the painter's brush to draw long fluid lines which take advantage of the quality of mark obtained with a full brush. The non-specific situation covers the area of impressionistic and non-representational drawing and painting. The order of testing was Experiments 4, 1, 2 and 3. A different original head was used with every test.

Ten right-handed subjects in their 1st term of the Batchelor of Arts drawing course at Camberwell College of Arts London volunteered for eye tracker testing. Their ages ranged from 19 to 47 (average 27), six were female and none wore spectacles or contact lenses. All had been drawing more or less frequently since childhood or secondary school days. The best way to describe the group is as comprising skilled amateurs with some experience in portrait painting and drawing and accustomed to being watched while at work. Subjects were informed of the experiments in general terms only and gave written consent to the tests which had the approval of the local ethical committee.

The eye tracker apparatus used was the head-mounted ASL 501 running at 50 Hz. Head position was monitored using an Ascension Flock of Birds magnetic tracker, the integrated system providing accuracies better than 1°. The scene in front of the subject was video recorded with a separate scene camera operating at 25 frames per second on a fixed tripod situated about 40 cm to the left of the subject's head. Sound was also recorded to capture the experimenter's instructions and for any subsequent conversation. The fixed camera position facilitated comparison between tests and subjects. The video recording provided a filmed image of the drawing hand and line in progress with superposed gaze position. During the analysis stage this image could be examined frame by frame in conjunction with the corresponding eye data supplied by the eye tracker. Each video frame being the result of two interlaced image scans, gaze position could be checked at the sampling rate of 50 Hz. Most importantly, this system allowed the visual record of a test to be consulted and analysed further at any stage in time. A fixation was identified when the point of gaze remained continuously within an area covered by a 1° visual angle for a minimum of 60 msec.

A nine-point calibration test was performed before each test and a “wand test” followed tests for which calibration accuracy required confirmation. In the wand test a technician moved smoothly by hand a 3 mm diameter marker fixed at the end of a thin rod along the line that had just been drawn, the subject having been instructed to follow this target with their eyes. The purpose of the exercise was to check that calibration was providing correct fixation positions when the subject was known to be foveating along the precise line that had just been drawn.

Testing procedures were planned in such a way as to record a subject's spontaneous response on hearing for the first time the experimenter's instructions formulated in the simplest possible terms. Subjects learned for the first time that they had to copy the heads half-way through Experiment 4. At that point they were made to understand that they should draw as precisely as possible. It was explained that even slight variation of shape and size should be accurately reproduced. Subjects were not asked to fixate a particular starting point. Instead, the eye tracker data and scene camera were switched on early, and subjects were allowed to find their natural way of beginning the drawing.

## 3 Experimental results

To facilitate comparisons between the different experiments, the detailed descriptions which follow all pertain to a same subject, AG. Unless specifically mentioned, other subjects performed with similar results. Table 2 provides results for AG as well as the mean results for all 10 subjects.

### 3.1 Experiment 1: Direct copying

In Experiment 1 the original and copy were placed side by side and were visible throughout the test. Subjects were free to choose appropriate eye and hand movements in a way that was constrained only by the task requirements. All subjects drew the heads in one continuous clockwise movement, the hand moving without interruption (except when drawing the eye) although with variations in speed, while gaze alternated rhythmically between the original and the pencil tip or its immediate vicinity. When drawing the eye the pencil had to be lifted from the paper and seven out of the 10 subjects then undertook two or more fixations on the original feature before resuming drawing. In general, the subject's head remained stationary and gaze shifts were produced essentially by eye movement alone.

**Fig. 1:**
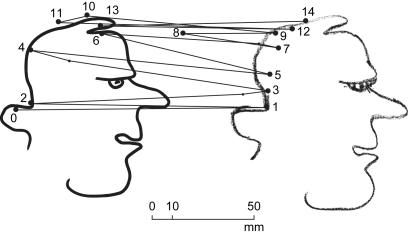
Direct copying. An example from Subject AG. Original on the left, copy on the right. Only fixations relating to drawing the back of the head are shown.

**Fig. 2:**
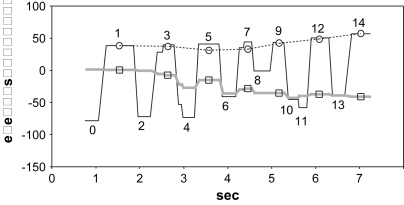
Eye position for Direct copying. Subject AG drawing back of head. Numbers are fixations as in Fig. 1. Black line is horizontal eye position, grey line is vertical eye position. Circles and dotted line are approximate horizontal pencil position; square is corresponding vertical pencil position.

Typically, the hand started drawing a segment of line at 1 with a stable fixation on the corresponding segment of the original line at 2 (Fig. 1). The hand then continued drawing while the fixation changed to the pencil at 3. Finally the fixation reverted to the next point on the original at 4 while the hand continued drawing. In this way fixation points on, or near, the drawn line were also indications of the pencil's progress (Fig. 2). The entire drawing was accomplished by Subject AG in 59 sec at an overall drawing speed of 6 mm/sec (AG was one of the slower subjects). Average “dwell times”, defined as the period during which a fixation, or series of contiguous fixations, remain either on the original or on the copy, were for Subject AG .326 sec on the original and .635 sec on the copy.

Of direct relevance to our analysis was the proportion of dwell time spent on the original relative to the total, i.e., to the sum of dwell time spent on original plus copy. This was 34% for AG and varied between subjects from 34% to 62% (mean 46%). This indicates that a very appreciable amount of drawing took place “blind” when the eye was on the original.

The rhythm of gaze movements between original and copy may be characterised by a “cycle” or average time elapsed between two consecutive gazes to the original. A cycle was measured as the quotient of the total test time divided by the number of saccades to the original. Total test time was inclusive of inter-dwell durations occurring during gaze shifts. The mean cycle for AG was 1.31 sec providing a rhythm of 46 cycles/min. The mean cycle value for all 10 subjects was 1.10 sec (56 cycles/min), ranging between .83 sec (72 cycles/min) and 1.34 sec (45 cycles/min).

Examined in detail, the overall rhythm of the eye–hand interaction pattern varied as the drawing progressed. At sections that were easy to draw, such as the back of the head, the hand moved faster and the distance between fixations on the original was greater. Fixations on the original could at times be ahead of the hand as, for example, in the case of fixation 4 located further along the original line than the corresponding pencil position between 3 and 5 (Fig. 1). At sections that were difficult to draw, such as the eyebrow and eye, the hand's speed decreased and could halt altogether as mentioned previously. Occasionally, saccades were observed to bring fixations onto previously drawn sections which, resumably, were acting as reference to the drawing action.

The overall standard of copying was high when evaluated on the amount of detail correctly reproduced. Thus in Fig. 1 it can be observed that the slight inflexions between points 2 and 4, 10 and 11, at the forehead and at the upper and lower lips were correctly replicated. The head's overall scaling and the relative proportions of individual features were also correctly rendered.

### 3.2 Experiment 2: Direct Blind copying

**Fig. 3:**
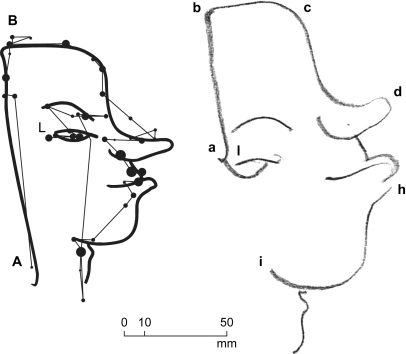
Direct Blind copying. Subject AG. Fixations on the original (left) while subject was drawing blind (right). Dot size proportional to fixation duration between .20 sec (smallest) to 2.48 sec (largest). Note progressive increase of drawing size. Letters are points where eye and hand arrival times were compared – see Fig. 4 (left).

A different original was used at every change of test. In Experiment 2 subjects drew blind on a horizontal pad held on their lap while looking at the original on the easel. All drew the contour in a continuous clockwise motion, slowing down at points of change in direction such as b, d and h, and interrupting only when the pencil had to be lifted from the paper to start a separate line segment (Fig. 3). Fixations were located generally on, or very close to, the original line. A first fixation “A” was made to position the hand at “a”. The hand started drawing “ab” when the eye was at “B”, and hand and eye terminated together at “l” and “L”. The entire drawing was accomplished in 40 sec at an average drawing speed of 9 mm/sec. Mean fixation duration was .825 sec.

**Fig. 4:**
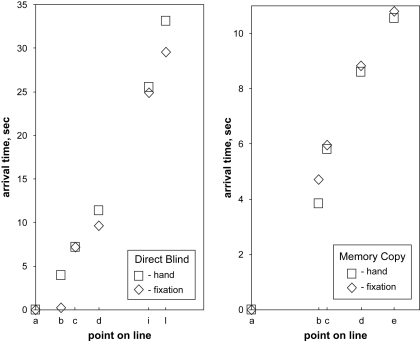
Eye and hand timing. Subject AG. Comparison of eye and hand arrival times at specific points of the drawing. Left: Direct Blind copying (see Fig. 3). Right: Memory copying (see Fig. 5).

Superposition of synchronous video recordings of the eye position on the original with the separately filmed hand position on the copy provided comparison of their relative positions. Three sections (ab, bc and hi) were drawn while fixations were at the corresponding end points, a behaviour not unlike the target locking mode observed when drawing simple lines to a given virtual point (Tchalenko, 2007) albeit in the present case the hand was hidden from view. Preliminary measurements on the superposed video timelines showed that these and other key points of the drawing were reached by the eye ahead of the hand by between .50 sec and 3.50 sec (Fig. 4 left). Although precise values of this time difference will need to be established with dedicated instrumentation and testing, the essential point to retain at this stage is that perception of the original and drawing of the copy were taking place simultaneously and that the action was lead by the eye.

The shapes of the head's individual components – back of the head, forehead, chin, neck, were reproduced with good accuracy. In contrast, the relative proportions between individual components were inconsistent, with line segments at the beginning of the drawing rendered too short and, at the end of the drawing, too long. With AG, as well as with all the other subjects, this resulted in the back of the head starting too small and the chin ending too big. The reason for this scaling error is unknown. Features such as the lower lip, eyebrow and eye which had required lifting of the hand from the paper, were systematically misplaced.

### 3.3 Interpretation of Experiments 1 and 2

In the Direct Blind copying experiment drawing took place while the subject was looking only at the original. Our two principal observations were that perception of the original and drawing of the copy took place simultaneously and that shape was correctly rendered but spatial positioning was defective.

The fact that visual perception of the original and motor execution of the copy occurred simultaneously suggests that drawing proceeded from a visuomotor mapping of the original and not from an encoded image of the original. It would in fact be difficult to comprehend the advantage of replacing an ongoing percept with its encoded image. This visuomotor mapping resulting in correct shape but defective spatial positioning fulfils the requirements of the Drawing Hypothesis and its corollary as defined in Section 1.

In the Direct copying experiment subjects spent between one and two thirds of the drawing time looking at the original and not at the copy. We make here the assumption that during these periods drawing took place as in the Direct Blind copying case, i.e., by a visuomotor mapping process based on the original and resulting in correct shape rendering. The rest of the drawing time was spent looking at the copy and this is, presumably, the reason for correct spatial positioning. With the above assumption, Direct copying can also be considered as fulfilling the requirements of the Drawing Hypothesis and its corollary.

### 3.4 Experiment 3: Memory copying

**Fig. 5:**
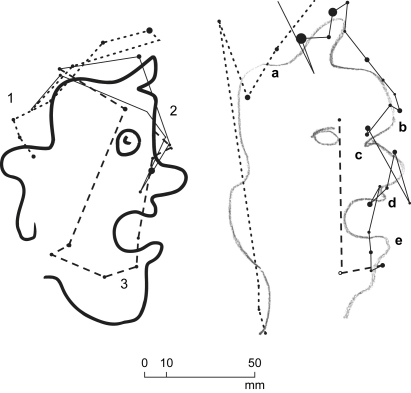
Memory copying. Subject AG. Encoding fixations on original (left) and drawing fixations on copy (right). Dot size proportional to fixation duration between .16 sec (smallest) to 1.76 sec (largest). Subject was drawing without seeing original. Numbers and line symbols indicate fixation passes during encoding (left) and drawing episodes during drawing (right). Letters are points where eye and hand arrival times were compared – see Fig. 4 (right).

Subjects perceived the original, knowing in advance that they would be copying it from memory. During the memorizing phase, AG examined the original contour line in three consecutive passes, producing three overlapping fixation paths: from ear to hair quiff, from quiff to nose and from jaw to nose. The first two passes were clockwise, the third, anticlockwise (Fig. 5). After each pass, the eye returned to a position near to its starting point. In contrast to Experiments 1 and 2, fixations were only approximately positioned with respect to the original line. Subjects appeared to be examining the line's details in both clockwise and counter clockwise order but were not systematically tracing the line as previously observed in Direct Blind copying.

Memorizing strategies varied to some extent between subjects. For example, one subject used a single clockwise path to cover the entire line, and another used three rapid consecutive clockwise paths each covering the entire line. We filmed separately throughout the test the subject's hand resting on their lap. Interestingly, with two of the subjects, a slight movement of the hand, as if in response to eye movements, was recorded during the memorizing phase.

Drawing proceeded clockwise with an irregular movement of the hand which, in AG's case, slowed down at times to complete standstill with the effect of subdividing the drawing action into five shorter episodes: back of head, quiff and forehead, nose and mouth, bottom lip and neck, and eye. With the exception of the first saccade to the top of head and the last saccade to the eye (the latter having been positioned separately at the end of drawing), the hand preceded the eye in a close pursuit type of movement. At four key points where comparison of eye and hand arrival times could be measured with confidence, the hand was ahead of the eye by up to .25 sec (Fig. 4 right, points b–e). Although this result was clearest with AG and less evident with some of the other subjects, the important observation was that the characteristic jagged appearance of the fixation path criss-crossing the head's outline and first observed during memorizing was repeated during drawing with all subjects.

Drawing speeds were about twice as fast as in Experiments 1 and 2, AG accomplishing the drawing in 26 sec at an overall speed of 19 mm/sec. Mean overall fixation duration on the original was .361 sec and on the drawing .527 sec. Copying accuracy with AG was average. The head's overall size and component proportions were broadly respected. Shape of individual facial components was correct but drawn without much detail. The same observation held for the other subjects, four of which drew heads slightly smaller than the original.

### 3.5 Experiment 4: Non-specific Memory copying

This test was performed before all the other tests and before subjects found out that they would be drawing heads. A sequence of heads was first memorized for the purpose of recognizing a repeat. When the repeat was presented subjects recognized it instantly in less than 1 sec. The repeat was then replaced with a blank paper, an operation which took about 3 sec. It was only then that subjects were instructed to draw the head from memory. All subjects found this to be a difficult exercise.

**Fig. 6:**
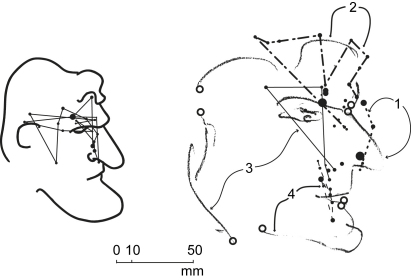
Non-specific Memory copying. Subject AG. Encoding fixations on original (left) and drawing fixations on copy (right). Subject was drawing without seeing original. Numbers and different fixation and saccade symbols represent order in which segments were drawn.

The memorizing fixation patterns obtained were radically different from those obtained with the previously described memory test. For all heads of the sequence the first fixation was located on the eye or in its immediate vicinity. Following this, 50% or more of the fixations remained located in this central region, the rest occurring during rapid forays to the regions of individual features – ear, base of hair and nose – only exceptionally falling on the original line itself (Fig. 6).

The drawing method was also different, with individual features put down in unconnected segments rather than as a continuous line, and in an anticlockwise order. For AG this was nose/mouth, forehead/top of head, ear/back of head, eyebrow/eye and chin/jaw. Fixations were unrelated, or only very loosely related, to the line being drawn. The entire drawing was accomplished in 17 sec at an overall drawing speed of 21 mm/sec. Mean overall fixation duration on encoding was .340 sec and on the drawing .370 sec.

Reproduction of shape accuracy was low in the case of Subject AG and extremely low with the other subjects who found it difficult to remember all the main features. In spite of this, something of the caricature aspects of the facial components had been retained in what was remembered: waviness of the hair, long downward direction of the nose, strangeness of the eye and protrusion of the chin. Note how AG corrected the chin, making it more protruding although this did not improve its shape compared to the original. Spatial positioning was average as regards the succession of components but defective as to overall size.

### 3.6 Interpretation of Experiments 3 and 4

The way of encoding observed in Experiment 3 was by fixation passes following only approximately the original line and resulting in a characteristic jagged pattern. Drawing then took place in short segments, with the hand generally leading the eye and pausing between segments, and a jagged fixation pattern similar to the one observed for encoding.

The similarity between the two fixation patterns prompts one to envisage encoding as a rehearsal by simulation of the drawing action. Jeannerod (1995, 2001) postulated a similarity in neural terms between the state where an action is simulated and the state of execution of that action. Because in drawing the execution state entails both eye and hand movements, simulation during encoding should likewise involve both the eye and the hand. Jeannerod proposed that this was indeed what happened, with both visual and motor systems being activated, the latter together with an inhibitory mechanism preventing actual muscular activity. Systematic tests would need to be devised to confirm more strongly this interpretation in the copying case.

Experiment 4 showed that encoding took place via a cluster of central fixations near the eye and regional fixations corresponding to the head's principal components. This radically different fixation pattern is not surprising as we know since Yarbus (1967) that fixation locations are consequent on the visual task which, in this test, is one of facial recognition, not of drawing. Facial recognition requires encoding the aspect of noteworthy features whereas, in the case of copying, drawing requires visuomotor mapping of a line. Our results conform to those of other facial recognition investigations: first fixations were observed to be systematically located in the region of the eye as found by Manor et al. (1995) using an abstract shape resembling an idealised face and containing and eye-like feature; subsequent fixations showed marked concentration in the ‘internal region’ covering the eyes, nose and mouth (Walker-Smith et al., 1977; Stacey et al., 2005) albeit in our case heads were seen in profile rather than in the conventional frontal view. These eye movements in the internal region during encoding are thought to help achieve high levels of recognition performance (Henderson et al., 2005). It was not possible to know from this experiment alone how accurate a mental image had been stored. However, the point of interest resided in the fact that the mental image was appropriate for instant recognition, yet not adequate for proper drawing from memory. It must be assumed that in the Non-specific Memory tests the required information for drawing had not been stored in memory, hence that shape of the line could only be drawn very approximately. McMahon (2002) has in fact suggested that drawing one's mental imagery is altogether impossible as this would require perception of one's imagery simultaneously with perception of the drawing, and hence would deploy simultaneously the same mental processes for two different goals.

Seen together, Experiments 3 and 4 demonstrate that the process of drawing from memory is unlikely to be one of simply encoding a visual image and subsequently performing on it a visuomotor transformation at the time of execution.

## 4 Discussion

### 4.1 Eye–hand interaction strategies

From the behavioural point of view, our experiments show that the task of copying a complex line varies with the drawing conditions, in particular with those concerning visibility of the copy in direct tests and of the original in memory tests. Four different eye–hand strategies have been documented.

In Direct copying where both the original and the copy are visible, the hand draws the copy line in one continuous movement while the eye alternates rhythmically between the pencil and the corresponding segment of the original. On the copy both shape and spatial positioning are accurate.

In Direct Blind copying where the original is visible but the copy hidden, the unseen hand draws the copy line in one continuous movement while the eye moves along the original line. The eye leads the drawing movement in target locking mode. On the copy shape is accurate but spatial positioning is size-deficient.

In Memory copying the original line is first memorized for the purpose of drawing and then hidden. The copy is visible throughout. During encoding, the eye covers the original in one or several rapid passes with fixations located only approximately on the line. During execution the hand draws the copy line in consecutive segments. The hand leads the drawing movement in close pursuit mode. Fixation patterns for encoding and execution are similar. On the copy shape reproduction is average but not detailed, and spatial positioning is average.

In Non-specific Memory copying a sequence of original images is first memorized for the purpose of facial recognition and then hidden after recognition has taken place. Only then is the subject told to draw the recognized face. The copy is visible throughout. During encoding, fixations are concentrated in a central region away from the original line. During execution, the hand draws the components individually, with the eye only very loosely connected to the hand's position. On the copy both shape and spatial positioning accuracies are low to very low.

### 4.2 The Drawing Hypothesis and just-in-time strategy

Instances when the conventional interpretation of a visuomotor transformation applied to an encoded visual mental image did not adequately describe drawing from life were mentioned in Section 1. We postulated an additional way of drawing governed by a different eye–hand interaction principle referred to as the Drawing Hypothesis and formulated as follows: the drawing of shape is the result of visuomotor mapping that can be executed directly while perceiving the original and without vision of the drawing surface. The corollary to this hypothesis would be that correct spatial positioning on the paper requires vision of the drawing surface. Our observations during Direct Blind tests where subjects only perceived the original confirmed this interpretation. Direct tests, where subjects perceived the original during about 46% of the time, also corroborated the Drawing Hypothesis providing the reasonable assumption was made that during this time the eye–hand behaviour was similar to that of the blind experiment. In these two drawing situations the Drawing Hypothesis is the better interpretation of observed eye and hand movements.

Drawing from memory, although not specifically covered by the Drawing Hypothesis, provided some useful additional observations. First, Memory copying tests were observed to involve a rehearsal process during encoding. Second, Non-specific Memory tests in which subjects encoded for recognition purposes were observed to be a poor basis for drawing. Together these tests suggest that the process of drawing from memory is unlikely to be one of simply encoding a visual image upon which is subsequently performed a visuomotor transformation at the time of execution.

The Drawing Hypothesis has also been supported by the accompanying work using functional brain imaging (Miall et al., 2009), in which brain activation levels were measured during the encoding phase and the drawing phase of a task directly comparable to the Direct Blind copying and Memory copying tasks reported here. In that work, activation patterns were consistent with visuomotor mapping during the encoding phase, and no evidence for retention and recall of a mental visual image was found.

Research in other tasks involving extraction of visual information in the service of specific behavioural goals has shown that subjects adopt a just-in-time strategy to minimize the use of working memory. They accomplish this by referring back to the source of information just in time for the intended action to proceed (Ballard et al., 1992, 1995, 2003; Hayhoe et al., 1998; Land, 2006). A similar behaviour has been reported in visual scene-comparison tasks by Gajewsky and Henderson (2005). The Direct copying task involves a continuous motion of the hand with a continuous back and forth motion of the eye, during which the eye spends on average nearly as much time on the original as on the copy. Both the original and the copy act as sources of information – the original essentially for shape and the copy essentially for spatial position. The overall behavioural mechanism can be considered as a double just-in-time strategy which minimizes, or avoids altogether, the use of working memory.

Further investigations will show whether the Drawing Hypotheses, seen to work in Direct and Direct Blind copying situations, can also be considered as an alternative way of drawing from life in more general real-world situations.

## Figures and Tables

**Table 1:** The two ways of drawing

Conventional	Drawing Hypothesis
Looking at original a. Original perceived b. Original encoded to visual memory Turn to paper c. Mental visual image perceived d. Line to be drawn decided e. Visuomotor mapping f. Line executed on paper	Looking at original a. Original perceived b. Line to be drawn decided c. Visuomotor mapping (allowing immediate rendering of shape) Turn to paper d. Line executed on paper

**Table 2:** Eye–hand parameters for Experiments 1–4

	Direct		Direct Blind		Memory		Non-specific Memory	
All	AG	All	AG	All	AG	All	AG
Mean fixation on original (sec)	.235 (.120)	.247 (.106)	.762 (.183)	.825 (.579)	.335 (.075)	.361 (.269)	.349 (.211)	.340 (.259)
Mean dwell on original (sec)	.374 (.158)	.326 (.101)						

Mean fixation on copy (sec)	.283 (.211)	.361 (.254)			.470 (.133)	.527 (.401)	.473 (.170)	.370 (.334)
Mean dwell on copy (sec)	.442 (.211)	.635 (.302)						

Dwell cycle duration (sec)	1.10	1.31						

Approx. drawing speed (mm/sec)	9	6	12	9	20	19	18	21

Standard deviations shown in parenthesis. Dwell and cycle durations defined in the text.
